# Do Studies on Cortical Plasticity Provide a Rationale for Using Non-Invasive Brain Stimulation as a Treatment for Parkinson’s Disease Patients?

**DOI:** 10.3389/fneur.2013.00180

**Published:** 2013-11-06

**Authors:** Giacomo Koch

**Affiliations:** ^1^Non-Invasive Brain Stimulation Unit, Neurologia Clinica e Comportamentale, Fondazione Santa Lucia IRCCS, Rome, Italy; ^2^Stroke Unit, Policlinico Tor Vergata, Rome, Italy

**Keywords:** Parkinson disease, transcranial magnetic stimulation, cortical plasticity, LTP and LTD, motor cortex

## Abstract

Animal models of Parkinson’s disease (PD) have shown that key mechanisms of cortical plasticity such as long-term potentiation (LTP) and long-term depression (LTD) can be impaired by the PD pathology. In humans protocols of non-invasive brain stimulation, such as paired associative stimulation (PAS) and theta-burst stimulation (TBS), can be used to investigate cortical plasticity of the primary motor cortex. Through the amplitude of the motor evoked potential these transcranial magnetic stimulation methods allow to measure both LTP-like and LTD-like mechanisms of cortical plasticity. So far these protocols have reported some controversial findings when tested in PD patients. While various studies described evidence for reduced LTP- and LTD-like plasticity, others showed different results, demonstrating increased LTP-like and normal LTD-like plasticity. Recent evidence provided support to the hypothesis that these different patterns of cortical plasticity likely depend on the stage of the disease and on the concomitant administration of l-DOPA. However, it is still unclear how and if these altered mechanisms of cortical plasticity can be taken as a reliable model to build appropriate protocols aimed at treating PD symptoms by applying repetitive sessions of repetitive TMS (rTMS) or transcranial direct current stimulation (tDCS). The current article will provide an up-to-date overview of these issues together with some reflections on future studies in the field.

## Introduction

Synaptic plasticity, in the form of long-term depression (LTD) and long-term potentiation (LTP), represents an intriguing mechanism that allows the encoding and retention of memories via the activity-dependent functional and morphological remodeling of synapses (1). Experimental models of Parkinson’s disease (PD) have consistently shown that dopamine plays a key role in the modulation of the altered mechanisms of synaptic plasticity detected in the basal ganglia (2, 3). In particular there is evidence suggesting a direct link between the degeneration of the substantia nigra and the impairment of neuroplasticity (3): the impairment in LTD and LTP induction is paralleled by dopamine depletion and is related to the symptoms onset (2, 4). Consistently, treatment with dopamine is also able to restore LTP expression (5). Another form of synaptic plasticity, named “depotentiation,” which results from the reversal of established LTP by a low-frequency (LF) stimulation protocol, was also found to be dependent on dopaminergic signaling and, interestingly, to be lost selectively in an experimental model of l-DOPA-induced dyskinesia (LID) (5).

In recent years an emerging amount of work was aimed at investigating these fascinating processes *in vivo*, directly in patients affected by PD. For instance, Prescott et al. (6) recorded evoked field potentials straight in the substantia nigra pars reticulata of PD patients undergoing therapeutic implantation of deep brain stimulating electrodes in the subthalamic nucleus. In these patients high-frequency (HF) stimulation did not induce a lasting change in field potential amplitude in the OFF state. The administration of l-DOPA potentiated the field potential amplitudes (LTP), providing an important evidence that PD patients have DOPA-dependent impaired mechanisms of LTP in the basal ganglia circuits.

## Cortical Plasticity in PD Patients

In PD patients, these forms of altered synaptic plasticity have been investigated more extensively in the primary motor cortex (M1) using various protocols of non-invasive brain stimulation (NIBS) such as paired associative stimulation (PAS) (7) and theta-burst stimulation (TBS) (8). PAS- and TBS-related changes in corticospinal excitability, as indexed by the increase or decrease in the motor evoked potential (MEP) amplitude, are thought to reflect respectively LTP-like or LTD-like phenomena (8). These methods have been used in the recent past to study the profile of M1 cortical plasticity in patients with PD in different experimental conditions (9–18).

## PAS Protocol

Paired associative stimulation take advantage of the principles of associative plasticity by repeatedly coupling a peripheral afferent input from the median nerve with a cortical TMS pulse applied over M1 with an inter-stimulus interval of 10–25 ms (7). This protocol normally decreases or enhances M1 excitability for at least 1 h, resembling mechanisms of Hebbian-like LTP or LTD mechanisms (7, 19–22). Early studies in patients with PD have reported abnormally reduced responses to PAS, compared with healthy subjects, pointing to a decreased cortical associative plasticity (10, 11). For instance, in a seminal study, Morgante et al. (10) showed that PAS significantly increased MEP size in controls but not in patients in OFF medication. l-DOPA restored the potentiation of MEP amplitudes induced by PAS in the non-dyskinetic group but not in the dyskinetic group. In contrast, another study reported as opposite enhanced responses to the same PAS protocol in PD patients (9). Recent evidence shed further light to these apparently contrasting results, by applying systematically PAS in both hemispheres in a large sample of “*de novo*” PD patients characterized by common asymmetric symptoms (23). LTP-like plasticity was more impaired in the “more affected” hemisphere, while there was an *increased* cortical associative plasticity in the “less-affected” hemisphere, thus suggesting compensatory functional sensorimotor reorganization in the early phase of PD (23). A debated question is whether these alterations of cortical plasticity are dependent on the underlying pathology or on the history of medication with l-DOPA. In a recent study Kacar et al. (24) aimed to address this issue by comparing responses to facilitatory PAS in two cohorts of advanced PD patients: one included chronically and optimally treated patients while the other group included patients with advanced PD who had never taken dopaminergic drugs. Again, the facilitatory responses to PAS were reduced in both cohorts of chronically treated and drug naïve PD patients when compared with healthy subjects. Importantly, this study indicates that in advanced PD, cortical associative plasticity seems to be impaired regardless of a previous chronic exposure to l-DOPA.

## TBS Protocol

Similarly to what observed with PAS, TBS protocols failed to induce plasticity in patients with PD in most studies (12, 13). Suppa et al. (13) tested the effects of intermittent theta-burst stimulation (iTBS), a technique used to induce LTP-like plasticity in M1, in patients with PD, OFF and ON dopaminergic therapy, with and without l-DOPA -induced dyskinesias (LIDs). In these patients iTBS failed to increase MEP responses in all conditions, suggesting a lack of iTBS-induced LTP-like plasticity in M1 in PD regardless of patients’ clinical features. Similar results were obtained by another group (25). Kishore and colleagues tested in a sample of *de novo* PD patients the effects of the iTBS and the cTBS protocols, the latter known to induce LTD (8). In these *de novo* PD patients there was no plasticity for both protocols. Acute l-DOPA challenge did not improve plasticity in either M1 cortices, though motor signs of PD improved. Thus these findings showed that an early, severe, and bilateral loss of plasticity in M1 in *de novo* PD patients is a primary disease-related cortical dysfunction. However, these results were in contrast with another similar study in which iTBS was performed in a sample of PD patients in ON and OFF l-DOPA therapy. PD patients had similar increases in MEP amplitude compared to baseline over the course of 60 min. Changes in intracortical circuits induced by iTBS were also comparable in the different groups, showing that iTBS produced similar effects on cortical excitability for PD patients and controls (18). These apparent discrepancies might be due likely to the different stages of disease of the patients recruited in the different studies. In a related work Kishore et al. (17) tested more systematically the effects of different TBS protocols in both OFF and ON l-DOPA therapy conditions in different groups of advanced PD patients. These were stratified according to their motor response to l-DOPA into stable responders, fluctuating non-dyskinetics and fluctuating dyskinetics. In OFF, stable responders showed both types of plasticity, fluctuating non-dyskinetics had LTP but no LTD while fluctuating dyskinetics lost both types of plasticity. These data suggest that there is a gradual loss of chronic treatment benefit on plasticity, particularly for LTD, when motor complications develop. Moreover, an acute non-physiological dopamine boost seems to have a negative effect on cortical plasticity as disease advances. This loss of cortical plasticity with progression of disease may contribute to the pathophysiology of motor complications. Similar results were obtained in another study in which PD patients with and without LIDs were compared. PD patients without LIDs had normal LTP- and depotentiation-like effects when they took their full dose of l-DOPA, but there was no LTP-like effect when they were on half dose of l-DOPA (14). In contrast, patients with LID could be successfully potentiated when they were on half their usual dose of l-DOPA; however, they were unresponsive to the depotentiation protocol. These latter results suggest that depotentiation is abnormal in the motor cortex of patients with PD with LID and that their LTP-like plasticity is more readily affected by administration of l-DOPA than their clinical symptoms (14).

Taken together these recent studies on neuroplasticity in PD patients suggest that:

- The mechanisms of LTP-like and LTD-like cortical plasticity can be impaired since the early phases of the disease (16, 23);
- The response to the different plasticity-inducing protocols and to dopamine administration are not fixed but may vary with the disease progression and with onset of motor complications (14, 16).
- These alterations cannot be promptly restored by dopamine administration in all conditions (13, 16);
- The response to the plasticity-inducing protocols seem not to be strictly associated to the clinical improvement induced by dopamine administration (13, 16).

## NIBS as a Treatment for PD

As showed by the previous paragraph some NIBS methods are useful to assess cortical plasticity in PD patients. However, NIBS tools may also have a relevant clinical impact when applied repeatedly over several weeks. In the past years there have been quite a lot of published studies aiming at reducing motor impairment in PD by means of NIBS techniques such as repetitive TMS (rTMS). rTMS at frequencies of 5 Hz and higher can enhance motor cortex excitability (26) whereas lower frequencies rTMS (1 Hz and lower) can transiently depress cortical excitability (27). Several randomized controlled trials used rTMS to treat the PD motor symptoms [(28) for a review]. These studies are extremely numerous, but are characterized by a large heterogeneity of cortical targets, stimulation protocols, and patients’ populations (28). In general the sample size was small and clinical effects are unlikely to be detected because of insufficient power. This, together with the variability of patient profile (various pharmacological treatment, disease duration, severity, and type of motor symptoms) made the emergence of consensus for any stimulation procedures extremely difficult. In general, while M1 was the most frequently studied target, clinical efficacy has been more modest using this target compared to the supplementary motor area (SMA) target of which value have been emphasized by recently published large multicenter trials (29).

Some negative results have been reported in controlled trials based on repeated sessions of LF rTMS of M1 [i.e., Ref. (30)]. On the other hand, the majority of the studies have tested the effects of repeated sessions of HF rTMS of M1 in PD patients. Some of these studies supported some therapeutic value of HF rTMS of M1 in PD, showing a global improvement of UPDRS part III motor scores, especially regarding movement speed or also gait velocity, following the focal stimulation of M1 hand representation (31, 32) or the bilateral stimulation of a larger M1 area (33–35). Such improvement could be related to an increase in dopamine release, although these results also suggest the possibility of placebo effects (34, 36). HF rTMS has been also applied over the leg area of M1 and followed by 30 min of treadmill training over 4 weeks, resulting in an increased walking speed (37). On the other hand only a few studies have reported negative results of HF rTMS of M1 in PD (38, 39). Moreover, two recent studies tested the effects of repeated sessions of iTBS of M1, with some controversial results on the clinical changes induced by the stimulation (40).

A promising alternative approach seems to be provided by stimulation of the SMA (41, 42). In a multicenter trial, PD patients were treated with 5 Hz rTMS once a week, during 8 weeks. The first report (41) showed some improvement of the global UPDRS score, while the second (42) indicated that the clinical improvement was restricted to the symptoms of bradykinesia. A recent multicenter trial confirmed that rTMS of SMA can have some clinical impact. The authors found an improvement on the UPDRS scores following a prolonged protocol of weekly performed LF rTMS of SMA on motor symptoms of PD (29), but not with HF rTMS. Interestingly, rTMS of the SMA has also been shown able to improve LID (43, 44). At this regard, an alternative approach could be provided by cerebellar stimulation. Reduction of peak-dose dyskinesia for up to 4 weeks was described following repeated sessions of excitability-decreasing cTBS bilaterally delivered to the lateral cerebellum (45). The rationale for cerebellar stimulation arises from the possibility to modulate cerebello-thalamo-cortical circuits (17, 46, 47).

To sum up, the current literature on therapeutic trials of rTMS in PD patients is still ambiguous, and the search for the most effective protocol is still on its way. Moreover there is almost no evidence that the clinical improvement induced by NIBS could be related to a restoration of the altered mechanisms of cortical plasticity described above. The next large multicenter trials should be designed in order to take in account the inter-individual variability observed in PD patients regarding the profile of cortical plasticity and its modulation by dopamine (16). The effects of the different protocols might be stratified according to the different profile of LTP-like and LTD-like alterations. This could allow identifying eventual responders or non-responder to a specific protocol of NIBS.

## Conflict of Interest Statement

The author declares that the research was conducted in the absence of any commercial or financial relationships that could be construed as a potential conflict of interest.
